# O089: Recurrent transmission of group a streptococcus pyogenes (GAS) during surgery by a health care worker (HCW)

**DOI:** 10.1186/2047-2994-2-S1-O89

**Published:** 2013-06-20

**Authors:** C Landelle, P Lesprit, P Legrand, P Brehaut, D Ducellier, J-F Papon, E Allaire, E Girou, J-P Becquemin, C Brun-Buisson

**Affiliations:** 1 Unité de Contrôle, Epidémiologie et Prévention de l'Infection, CHU Albert Chenevier-Henri Mondor, Assistance Publique-Hôpitaux de Paris; Université Paris 12, Créteil, France; 2 Service de Bactériologie-Virologie-Hygiène, CHU Albert Chenevier-Henri Mondor, Assistance Publique-Hôpitaux de Paris; Université Paris 12, Créteil, France; 3 Service d’Oto-Rhino-Laryngologie, CHU Albert Chenevier-Henri Mondor, Assistance Publique-Hôpitaux de Paris; Université Paris 12, Créteil, France; 4 Service de Chirurgie Vasculaire et Endocrinienne, CHU Albert Chenevier-Henri Mondor, Assistance Publique-Hôpitaux de Paris; Université Paris 12, Créteil, France; 5 Service de Réanimation Médicale, CHU Albert Chenevier-Henri Mondor, Assistance Publique-Hôpitaux de Paris; Université Paris 12, Créteil, France

## Introduction

Surgical site infections (SSI) due to GAS are rare but potentially life-threatening.

## Objectives

We describe 2 cases occurring after thyroidectomy in two female patients (aged 36 and 52 years respectively), who developed septic shock with multi-organ failure, mediastinitis and empyema, respectively 2 and 4 days after surgery performed 4 months apart (Nov. 2009; Feb. 2010).

## Methods

We interviewed patients or relatives and operating room (OR) personnel with regard to recent throat infection, and investigated SSI prevention measures, surgical masks wearing in the OR, and GAS carriage by HCWs; GAS isolates were compared by molecular typing.

## Results

There was no recent history of throat infection in patients or their relatives. Compliance to SSI preventive measures (pre operative showers, skin antisepsis, and laminar air flow) was adequate, and wearing of mask in the OR was adequate for 88% of 332 HCWs. A GAS isolate was recovered from throat swabs of 2 of the 6 HCWs caring for the 1^st^ case, one of which was identical to the patient’s isolate. Auditing this HCW revealed a lack of adequate fitting of the mask during preparation of the OR. Educational sessions were implemented. After the 2^nd^ case occurred, the same HCW was again found colonized with a GAS isolate identical to the patient’s isolate, but different from the 1^st^ one. A more in-depth investigation revealed that one of his children had recurrent tonsillitis. Decolonization of the HCW was attempted, but GAS carriage recurred until tonsillectomy was performed on his child.

## Conclusion

The same staff carrier was involved in the transmission of 2 different GAS strains, likely resulting from household transmission. Throat carriage of the personnel stopped only after tonsillectomy of his child. Reinforcing adequate surgical mask wearing in the OR is important, but 100% compliance appears difficult to maintain.

## Disclosure of interest

None declared.

